# The relationship between patient height and intracranial pressure in children and adults

**DOI:** 10.1186/2045-8118-12-S1-P2

**Published:** 2015-09-18

**Authors:** Trine Hjorslev Andreasen, Sarah Skovlunde Hornshøj, Alexander Lilja, Morten Andresen, Marianne Juhler

**Affiliations:** 1 Copenhagen University Hospital Rigshospitalet, Denmark

## Introduction

The management of increased intracranial pressure (ICP) and monitoring of ICP is an important part of neurosurgery, but reference values for ICP have not been established differentiating between children and adults. The same estimated reference of 7-15 is used indiscriminately for children and adults, and does not take in to account that adults and children differ physiologically. The effect on ICP of a postural change from a supine to a upright standing position has been established, though the association between height and ICP in upright position has not been studied. The purpose of this study is to address if ICP values could depend on height.

## Methods

Patients having a shunt were excluded from the study in order to avoid the influence of mechanical siphoning. Forty-one patients undergoing diagnostic ICP monitoring for a suspicion of hydrocephalus or idiopathic intracranial hypertension (IIH) were included in this study. Data were consecutively and prospectively collected. Nine were children (age 7-17) and thirty-two were adults (age 23-85). ICP was measured in parenchyma by either a cable-based or a telemetric probe. Measurements included both a supine and a upright standing position, and changes in ICP were calculated.

## Results

Mean height was 152.3 cm (range 122-180) for children and 169.4 cm (range 155-188) for adults. Height did not appear to have an impact on measured ICP in either supine (p = 0.15) or upright standing position (p = 0.28). Changing body position from supine to upright caused a significant decrease in ICP in both children (median decrease 9.8 mmHg; p = 0.008) and adults median decrease 12.5 mmHg; p<0.001).

In the entire group there was borderline significance between patient height and the calculated postural decrease in ICP occurring when the patient changed from a supine to a upright standing position (p = 0.053). When dividing into children and adults, the change in ICP correlated to height was significant in children (p = 0.022), but not in adults (p = 0.34).

## Conclusions

We did not establish a direct correlation between ICP and height. However, in children we found a relationship between height and ICP decrease going from supine to upright. A similar correlation was not found in adults. Our results may be important for approaches to physiological risks of overdrainage caused by shunting.

